# Endocytosis and Transport of Growth Factor Receptors in Peripheral Axon Regeneration: Novel Lessons from Neurons Expressing Lysine‐Deficient FGF Receptor Type 1 *in vitro*

**DOI:** 10.1002/ar.24120

**Published:** 2019-04-22

**Authors:** Barbara Hausott, Alexandra Förste, Fabian Zach, Stefan Mangger, Ellen Margrethe Haugsten, Lars Klimaschewski

**Affiliations:** ^1^ Department of Anatomy, Histology and Embryology, Division of Neuroanatomy Medical University Innsbruck Innsbruck Austria; ^2^ Department of Tumor Biology Institute for Cancer Research, Norwegian Radium Hospital Oslo Norway; ^3^ Centre for Cancer Cell Reprogramming, Institute of Clinical Medicine, Faculty of Medicine, University of Oslo Oslo Norway

**Keywords:** sensory neuron, ERK, AKT, receptor tyrosine kinase, recycling

## Abstract

In the course of peripheral nerve regeneration, axons encounter different extracellular growth factors secreted by non‐neuronal cells at the injury site and retrogradely transported after binding to neuronal membrane receptor tyrosine kinases. The present study reviews the role of receptor transport in peripheral axon outgrowth and provides novel data on trafficking of fibroblast growth factor receptor type 1 (FGFR1). Differences in receptor transport are determined by different numbers of lysine residues acting as ubiquitination sites in the intracellular receptor domain. We previously demonstrated that overexpression of mutant FGFR1‐25R (25 out of 29 intracellular lysines replaced with arginine) results in enhanced receptor recycling as compared to wild‐type FGFR1 followed by strong stimulation of elongative axon growth *in vitro*. Here, the effects of lysine‐deficient FGFR1 (FGFR1‐29R lacking all 29 cytoplasmic lysine residues) or of only 15 lysine mutations (FGFR1‐15R) on axon outgrowth and concomitant changes in signal pathway activation were investigated by immunocytochemistry and morphometry of cultured primary neurons. Overexpression of FGFR1‐15R in adult sensory neurons resulted in enhanced receptor recycling, which was accompanied by increased axon elongation without stimulating axon branching. By contrast, FGFR1‐29R was neither endocytosed nor axon outgrowth affected. Although overexpression of FGFR1‐15R or FGFR1‐25Ra strongly promoted elongation, we did not detect increased signal pathway activation (ERK, AKT, PLC, or STAT3) in neurons expressing mutant FGFR1 as compared with wild‐type neurons raising the possibility that other signaling pathways or signaling independent mechanisms may be involved in the axon outgrowth effects of recycled FGF receptors. Anat Rec, 302:1268–1275, 2019. © 2019 The Authors. *The Anatomical Record* published by Wiley Periodicals, Inc. on behalf of American Association of Anatomists.

## INTRODUCTION

Peripheral nerve regeneration is strongly influenced by neurotrophic factors and cytokines (Klimaschewski et al., 2013). Following peripheral nerve lesion, growth factors are expressed mainly by Schwann cells and activate receptor tyrosine kinases (RTKs) present at the axonal plasma membrane. Neurotrophins such as nerve growth factor (NGF) or brain derived neurotrophic factor (BDNF) are released from dedifferentiating Schwann cells in the distal nerve stump after transection. They bind to their cognate high‐affinity receptors, are internalized by endocytosis, and are transported retrogradely *via* early and late endosomes. Since the active cytoplasmic receptor kinase domain is facing the cytoplasm, these endosomes stimulate intra‐axonal signaling pathways during their transport along considerable distances. Upon arrival in the cell body, signaling molecules may be released and regulate neuronal gene expression after import into the nucleus. Some RTKs shuttle back to the surface membrane, that is, they are recycled. These contribute significantly to elongative axon regeneration at least *in vitro* (Ascano et al., 2012; Klimaschewski et al., 2013).

Members of the fibroblast growth factor (FGF) family act as neurotrophic factors in the lesioned peripheral nervous system. For example, FGF‐2 is upregulated in response to a peripheral nerve lesion (Ji et al., 1995; Grothe et al., 1997; Klimaschewski et al., 1999; Grothe et al., 2001) and has been shown to promote peripheral axon regeneration *in vivo* (Danielsen et al., 1988; Aebischer et al., 1989; Fujimoto et al., 1997; Timmer et al., 2003). FGF receptor type 1 (FGFR1) is the most abundant FGF receptor in the nervous system (Ford‐Perriss et al., 2001). FGFR1 activation results in the induction of different intracellular signaling pathways such as the Ras/extracellular signal‐regulated kinase (ERK, see review by Hausott et al. in this issue), the phosphatidylinositol‐3 kinase (PI3K)/proteinkinase B (AKT), the phospholipase C (PLC), or the signal transducer and activator of transcription (STAT3) pathways (Mason, 2007).

**Figure 1:**
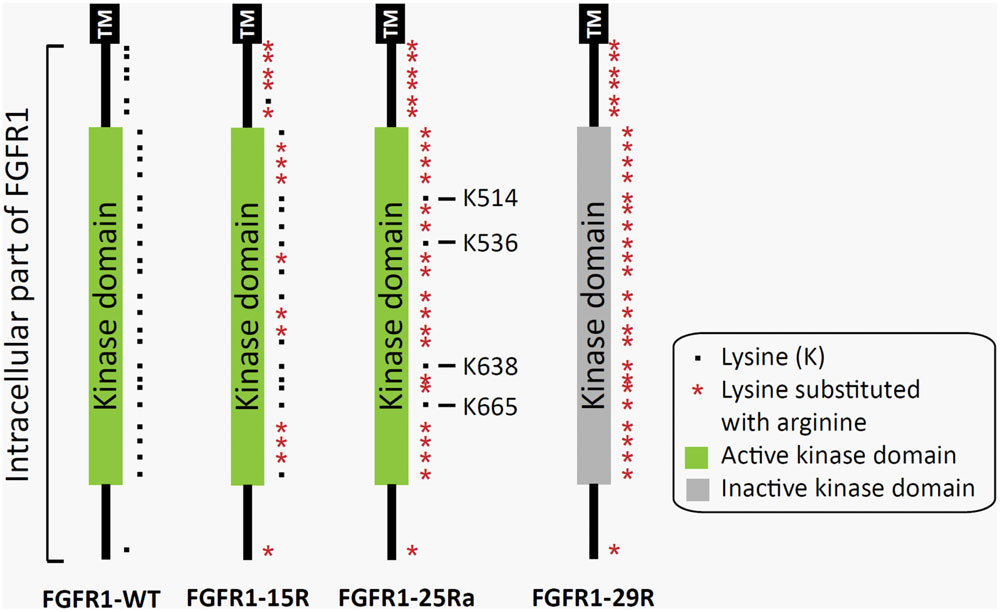
Scheme of the intracellular part of FGFR1‐WT and lysine mutants FGFR1‐15R, FGFR1‐25Ra, as well as FGFR1‐29R. Black dots indicate lysine residues and red asterisks indicate lysines substituted to arginine. A green kinase domain indicates an active receptor, whereas a kinase domain in gray an inactive receptor (FGFR1‐29R). Some important lysine (K) residues are labeled with their number. K514 is known to be crucial for receptor kinase activity and mutants in which K514 is conserved remain signaling active. Abbreviation: TM, transmembrane region.

FGFR1 is rapidly sorted to lysosomes for degradation, whereas FGFR4 is predominantly recycled back to the plasma membrane. The intracellular part of FGFR1 contains 29 lysine residues that function as potential ubiquitination sites for receptor degradation, while FGFR4 contains 16 lysine residues only (Haugsten et al., 2005). It was demonstrated that the mutation of up to 26 lysines in FGFR1 results in enhanced recycling of signaling active FGFR1 (FGFR1‐26Rc), whereas the mutation of all 29 lysines (FGFR1‐29R) abolishes receptor endocytosis as well as signaling (Haugsten et al., 2008). By contrast, signaling active mutants FGFR1‐15R and FGFR1‐25Ra (containing 15 and 25 mutated lysines, respectively) with conserved lysine 514 (K514) are internalized normally but rather sorted to recycling endosomes than to degradation in lysosomes (Fig. 1). Others observed a similar effect for mutants of FGFR3 with lower ubiquitination levels that exhibit enhanced recycling and slower rates of receptor degradation (Cho et al., 2004).

Our previous studies revealed that overexpression of FGFR1 promotes elongative axon growth of adult dorsal root ganglia (DRG) neurons *in vitro* (Hausott et al., 2008). This effect is further enhanced by treatment with the protease inhibitor leupeptin that prevents lysosomal degradation of FGFR1 and increases cell surface localization by stimulating receptor recycling (Hausott et al., 2012). Following overexpression of the lysine mutant FGFR1‐25Ra with enhanced recycling capabilities, the maximal axonal length (as an indicator of axon elongation) doubled within 24 hr while the number of axonal branch points remained unchanged.

**Figure 2:**
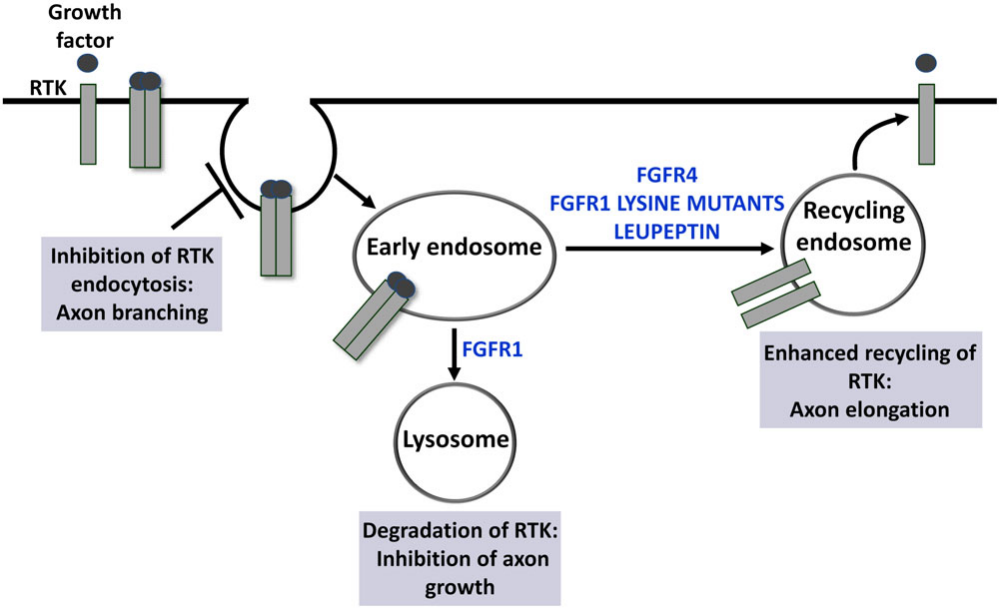
In response to growth factor activation, RTKs undergo endocytosis and are sorted to lysosomes for degradation or recycled back to the plasma membrane. FGFR1 is rapidly sorted to lysosomes for degradation, whereas FGFR4 is predominantly recycled (Haugsten et al., 2005). These differences in endocytic transport are determined by different numbers of lysine residues acting as ubiquitination sites in the intracellular domain (Haugsten et al., 2008). The protease inhibitor leupeptin enhances recycling of FGFR1 and promotes elongative axon growth of adult DRG neurons *in vitro*. Furthermore, lysine mutants of FGFR1 with enhanced recycling capabilities strongly promote elongative axon growth of adult DRG neurons (Hausott et al., 2008; Hausott et al., 2012). By contrast, inhibition of FGFR1 endocytosis inhibits axon elongation and enhances axonal branching (Hausott et al., 2011). Abbreviations: DRG, dorsal root ganglia; FGFR1, fibroblast growth factor receptor type 1; FGFR4, fibroblast growth factor receptor type 4; RTK, receptor tyrosine kinases.

These studies provide evidence for the relevance of enhanced RTK recycling for axon regeneration that was also demonstrated for the NGF receptor TrkA (Ascano et al., 2012). Furthermore, the neuronal cell adhesion molecule (NCAM) supports regeneration following nerve injury (Maness and Schachner, 2007; Zhang et al., 2008) and has been shown to promote recycling of FGFR1 (Francavilla et al., 2009b). By contrast, inhibition of FGFR1 endocytosis by methyl‐β‐cyclodextrin or chlorpromazine induced axon branching and impaired axonal elongation (Hausott et al., 2011). Together, these studies indicate the importance of intracellular trafficking of RTKs for axon growth and morphology (Fig. 2).

In the present study, we further investigated the effects of lysine mutants of FGFR1 on axon growth of adult sensory neurons and analyzed their signaling competence and intracellular trafficking as compared with wild‐type FGFR1 (FGFR1‐WT). We tested FGFR1‐15R (with only 15 lysine residues mutated to arginine) with enhanced recycling capabilities and compared it with the signaling‐deficient mutant FGFR1‐29R. FGFR1‐15R stimulated elongative axon growth of adult sensory neurons similarly to FGFR1‐25Ra but did not change levels of phosphorylated ERK (pERK) and phosphorylated AKT (pAKT) in cell bodies or axons as compared with FGFR1‐WT by immunocytochemistry. By contrast, signaling‐deficient mutant FGFR1‐29R did not promote axon growth and reduced the number of branch points.

## METHODS

### Cell Culture and Transfection

Adult rat DRG were dissected, collected in RPMI medium with antibiotic‐antimycotic, and treated with collagenase (5,000 units/ml) for 60 min followed by 0.25% trypsin/EDTA for another 15 min. They were then transferred to RPMI medium containing 10% horse serum and 5% fetal bovine serum and dissociated by 5–10 passages through a fire‐polished Pasteur pipette. Softened DRGs were electroporated using the Amaxa Nucleofector system (Lonza) with 3 μg of pcDNA3‐FGFR1‐WT or pcDNA3‐FGFR1 lysine mutants FGFR1‐15R, FGFR1‐25Ra, FGFR1‐26Rc, or FGFR1‐29R (Haugsten et al., 2008) plus 1 μg of enhanced green fluorescent protein (EGFP) for axon growth and signaling experiments. For trafficking and localization experiments, DRG neurons were transfected with 5 μg of pEGFP‐N1‐FGFR1‐WT or lysine mutants (FGFR1‐WT‐EGFP, FGFR1‐15R‐EGFP, FGFR1‐25Ra‐EGFP, or FGFR1‐29R‐EGFP). pEGFP‐N1‐FGFR1‐WT and lysine mutants were generated by PCR amplification using pcDNA3‐FGFR1‐WT or lysine mutants as template with the following primers: 5‐CAACTCGAGATGTGGAGCTGGAAG‐3 and 5‐GGCGGGCCCGGCGACGTCTGAGTCC‐3. The PCR product was cut with *XhoI* and *ApaI* and ligated into pEGFP‐N1 cut with the same enzymes. Transfected neurons were plated on glass dishes (WillCo) coated with poly‐D‐lysine (overnight) and laminin (4 hr). Cultures were maintained in RPMI medium with B27 supplement (Gibco Invitrogen) and antibiotic‐antimycotic at 37°C in a humidified atmosphere with 5% CO_2_.

### Measurement of Axon Growth

For measurement of axon growth, DRG neuron cultures cotransfected with FGFR1‐WT, FGFR1‐15R, FGFR1‐26Rc, or FGFR1‐29R plus EGFP were treated with FGF‐2 (100 ng/mL + 10 μg/mL heparan sulfate) for 24 hr. Transfected neurons were documented using an inverted fluorescence microscope (Zeiss Axiovert 100) equipped with a SPOT RT digital camera connected to a PC. The cells were first documented 48 hr after transfection and treated with FGF‐2, and after 24 hr of FGF‐2‐treatment, the same cells were documented again (recovered by *x*‐and *y*‐coordinates). Growth was determined within this 24‐hr period. MetaMorph morphometry software (Visitron Systems) was applied to measure the total axonal length, the maximal distance of the longest axon (parameter for axonal elongation), and the number of branch points. All morphologically intact neurons per dish with maximal distances ≥100 μm were analyzed.

### Immunostaining of DRG Cultures

For immunolabeling, neurons overexpressing EGFP alone or FGFR1‐WT, FGFR1‐15R, and FGFR1‐25Ra cotransfected with EGFP were treated with FGF‐2 (100 ng/mL + 10 μg/mL heparan sulfate; Sigma) for 30 min, 4 hr, 24 hr, or 48 hr and fixed for 15 min with 4% paraformaldehyde at room temperature. Cells were permeabilized with 0.5% Triton X‐100 for 10 min and blocked in 3% normal goat serum (in PBS) for 1 hr. Primary antibodies (all from Cell Signaling) raised against pERK (#9101, 1:400), ERK (#4696, 1:100), pAKT (#4060, 1:200), pPLC (#8713, 1:200), or phosphorylated STAT3 (pSTAT3) (#9131, 1:200) were applied overnight at 4°C and detected by secondary anti‐rabbit Alexa Fluor 546 (1:2,000, Molecular Probes). Average intensities of whole cell body fluorescence were measured for pERK, pAKT, and pPLC and of the nucleus for pSTAT3 following background correction applying the MetaMorph morphometry software.

### Quantification of Receptor Fluorescence and Colocalization with Transferrin

Dissociated DRG neurons overexpressing FGFR1‐WT‐EGFP, FGFR1‐15R‐EGFP, FGFR1‐25Ra‐EGFP, or FGFR1‐29R‐EGFP were treated with FGF‐2 (100 ng/mL + 10 μg/mL heparan sulfate) for 30 min to determine colocalization with recycling endosomes using transferrin, a marker for endocytic recycling. Cells were stained with transferrin conjugated to Alexa Fluor 594 (Molecular Probes) for 15 min. After washing, cells were fixed for 10 min with 4% paraformaldehyde at room temperature. Images were acquired with confocal‐like technology using a fluorescence microscope (Zeiss AxioObserver) equipped with an ApoTome slider. Single sections through the largest extent of the nucleus were chosen for analysis. For measurement of colocalization of FGFR1‐WT‐EGFP, FGFR1‐15R‐EGFP, FGFR1‐25Ra‐EGFP, and FGFR1‐29R‐EGFP with transferrin, digital image analysis was performed using the colocalization module of the AxioVision (Zeiss) software. The intensity threshold of a section was set for each channel separately using the histogram function. The threshold was set by multiplying the intensity of the cellular background by a factor of three. Threshold‐segmented objects were counted with the MetaMorph software including all objects with a minimum size of 9 and a maximum size of 45 pixels (pixel size of 0.1 × 0.1 μm^2^).

For measurement of subcellular receptor localization, DRG neuron cultures overexpressing FGFR1‐WT‐EGFP, FGFR1‐15R‐EGFP, FGFR1‐25Ra‐EGFP, or FGFR1‐29R‐EGFP were treated with FGF‐2 (100 ng/mL + 10 μg/mL heparan sulfate) for 30 min. Cells were fixed for 10 min with 4% paraformaldehyde at room temperature and images were acquired by ApoTome confocal‐like technology with the AxioVision software. Digital image analysis was performed to measure cell surface *versus* cytoplasmic distribution of FGFR1‐EGFP using MetaMorph. The mean average fluorescence intensity of the whole plasma membrane region (Fm) was calculated relative to the average intensity of three randomly selected cytoplasmic areas (Fc) after background correction.

For statistical analysis, one‐way ANOVA with Tukey's posttest was applied.

## RESULTS

### Axon Growth of Adult DRG Neurons Overexpressing FGFR1 Lysine Mutants

**Figure 3:**
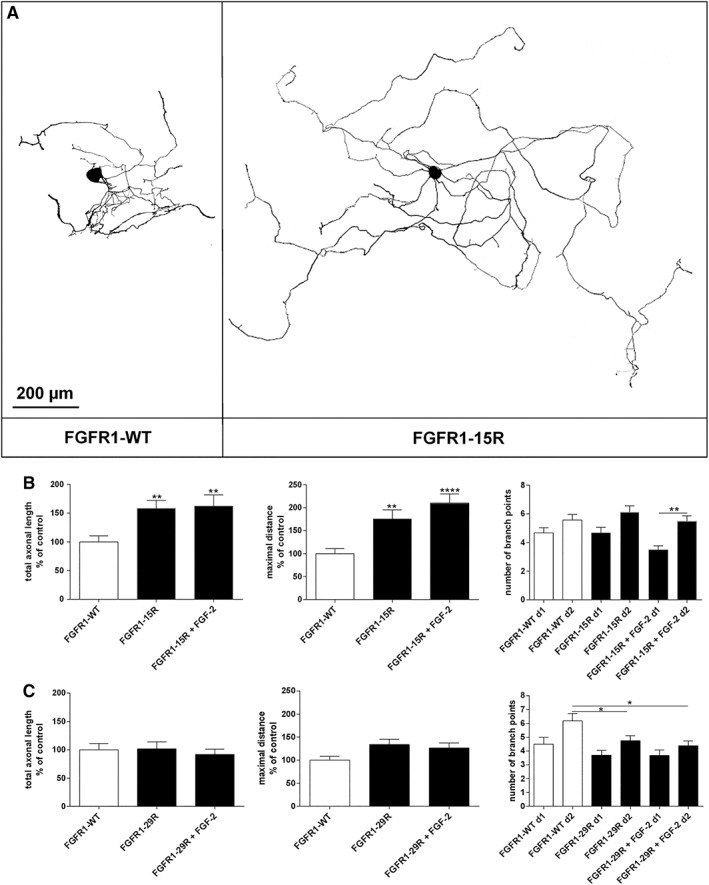
**(A)** Representative examples of neuronal morphologies in DRG cultures 72 hr after transfection with FGFR1‐WT or FGFR1‐15R and cotransfected with EGFP plasmid. Inverted fluorescence images are shown after cell body and background correction to document the different lengths of the axons. Axon growth of adult DRG neurons overexpressing **(B)** mutant FGFR1‐15R with enhanced recycling capabilities or **(C)** lysine deficient and signaling inactive FGFR1‐29R 24 hr after treatment with FGF‐2 as compared with FGFR1‐WT. Total axonal length, maximal distance of the longest axon, and number of branch points per cell were measured. The same cell was first analyzed 48 hr after transfection (d1), and then treated for 24 hr with FGF‐2 and documented again (d2). Growth was determined within this 24 hr period. One‐way ANOVA with Tukey's posttest (mean ± SEM of 4–5 independent experiments with a total number of neurons per group ≥50. *P < 0.1, **P < 0.01, and ****P < 0.0001 compared with FGFR1‐WT). Abbreviations: DRG, dorsal root ganglia; FGFR1‐WT, wild‐type fibroblast growth factor receptor type 1.

Our previous study demonstrated that overexpression of the lysine mutant FGFR1‐25Ra with 25 mutated lysines promotes elongative axon growth of adult DRG neurons (Hausott et al., 2012). This prompted us to investigate the effects of FGFR1‐15R comprising 15 mutated lysine residues to test our hypothesis that enhanced recycling but not the number of lysines is responsible for this effect. Overexpression of FGFR1‐15R increased elongative axon growth by adult sensory neurons obtained from rat DRGs (Fig. 3). The total axonal length and the maximal length of the longest axon were strongly enhanced compared with FGFR1‐WT with or without FGF‐2 indicating at least partial autoactivation of FGFR1‐15R upon overexpression. The maximal distance doubled with FGFR1‐15R plus FGF‐2, whereas axon sprouting was not different between FGFR1‐WT and FGFR1‐15R as revealed by the number of axonal branch points at 48 hr (d1) and 72 hr (d2) after plating. The graph reflects absolute values of axon branch points to indicate the stability of the culture between 48 and 72 hr after plating of the neurons. FGFR1‐26Rc with 26 mutated lysines promoted axon growth as well (data not shown). It is important to note that overexpression of FGFR1‐15R‐EGFP fusion protein did not reveal any effects on axon elongation or branching suggesting that the distal cytoplasmic part of the mutated receptor is highly relevant for the changes in axon growth (data not shown). By contrast, signaling inactive mutant FGFR1‐29R that does not contain any cytoplasmic lysine residues and does not undergo endocytosis (Haugsten et al., 2008) did not promote axon growth but revealed a reduced number of branch points as compared with FGFR1‐WT (Fig. 3). Thus, recycling of FGFR1 supported long‐distance axon regeneration *in vitro*, whereas signaling inactive FGFR1 that is not endocytosed had no effect on axon outgrowth.

### Signaling of FGFR1 Lysine Mutants in Adult DRG Neurons

**Figure 4:**
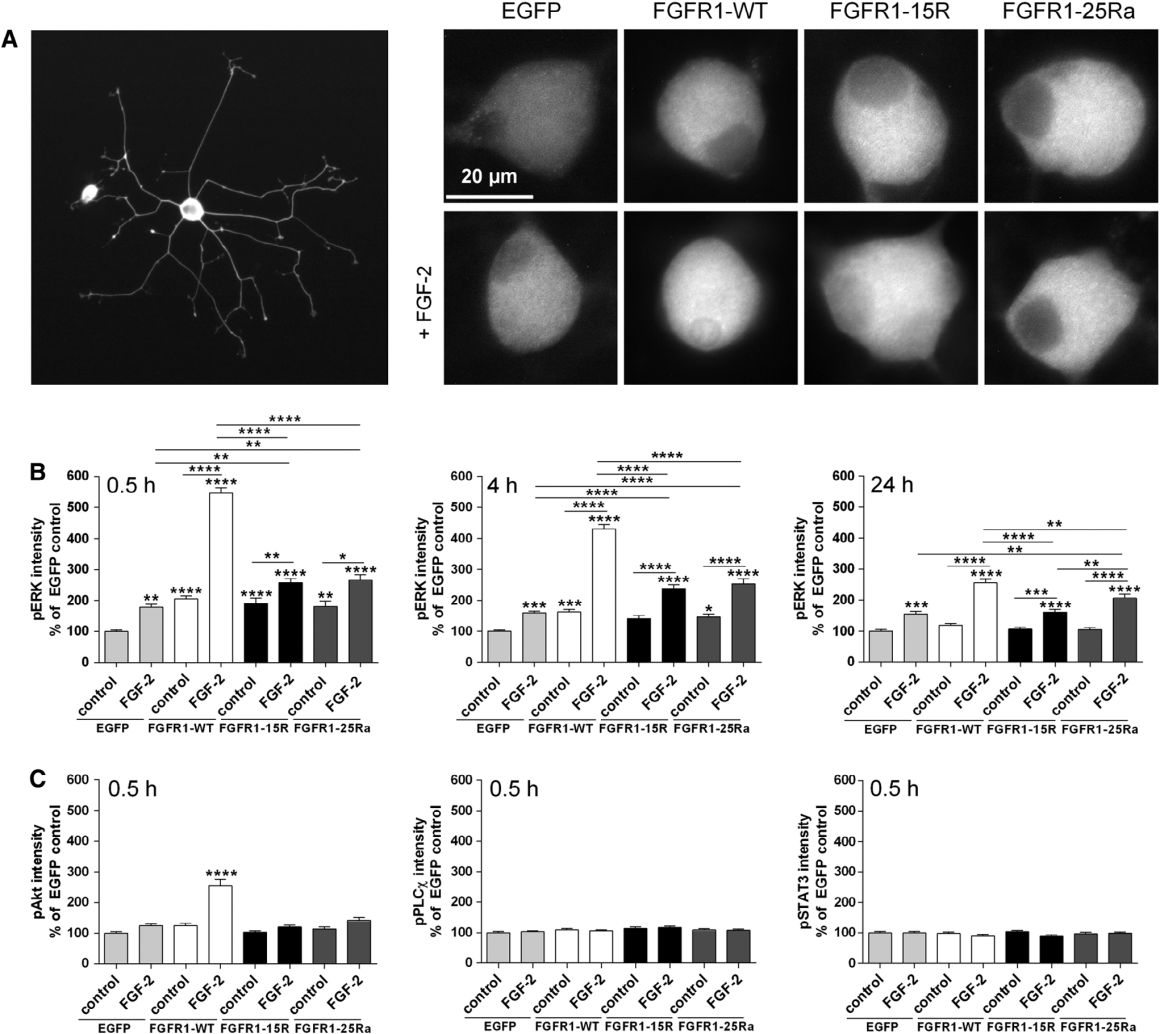
**(A)** Representative examples of DRG neurons overexpressing EGFP, FGFR1‐WT, FGFR1‐15R, and FGFR1‐25Ra after 30 min of treatment with FGF‐2 followed by fixation and fluorescent labeling with pERK antibodies. **(B)** Average fluorescence intensity of pERK, pAKT, pPLC, and pSTAT3 immunostaining of neurons overexpressing EGFP, FGFR1‐WT, FGFR1‐15R, and FGFR1‐25Ra after 30‐min, 4‐hr, and 24‐hr treatment with FGF‐2. One‐way ANOVA with Tukey's posttest (mean ± SEM of 3–4 independent experiments with a total number of neurons per group ≥70. *P < 0.1, **P < 0.01, ***P < 0.001, and ****P < 0.0001 compared with EGFP control). Abbreviation: DRG, dorsal root ganglia.

We then analyzed intracellular signaling pathway activation by FGFR1‐15R and FGFR1‐25Ra in DRG neurons by immunofluorescence, because Western blotting was not possible due to the low transfection rate. The 25Ra mutant significantly stimulated axon outgrowth over FGFR1‐WT as described before (Hausott et al., 2012). Surprisingly, pERK immunoreactivity after 30 min of FGF‐2 treatment was stronger in DRG neurons overexpressing FGFR1‐WT than in neurons with either FGFR1‐15R or FGFR1‐25Ra (Fig. 4A,B). Activation of ERK without FGF‐2 was similar in both mutants and in FGFR1‐WT overexpressing neurons and stronger than that in EGFP control transfected cells. Since pERK labeling was enhanced in FGFR1‐WT overexpressing neurons 30 min after FGF‐2‐treatment as compared with FGFR1 mutants, we tested whether the promotion of elongative axon growth induced by FGFR1‐15R and FGFR1‐25Ra was possibly caused by prolonged pERK signaling. However, ERK activation induced by FGF‐2 was always stronger in FGFR1‐WT than in FGFR1‐15R and FGFR1‐25Ra at 4 and 24 hr. After 48 hr of FGF‐2‐treatment, pERK was equally enhanced in FGFR1‐WT and FGFR1‐15R, whereas activation by FGFR1‐25Ra was still weaker than in FGFR1‐WT (data not shown). Immunostaining with antibodies against total ERK did not reveal differences between the different experimental groups (data not shown). Thus, FGF‐2‐induced ERK signaling in neurons overexpressing FGFR1‐15R or FGFR1‐25Ra was stronger than that in EGFP control transfected neurons but not exceeding pERK levels in FGFR1‐WT overexpressing neurons. We then analyzed levels of pAKT, pPLC, and pSTAT3, which have all been demonstrated to be involved in axonal growth as well. Interestingly, AKT activation after 30 min of FGF‐2‐treatment was only observed in FGFR1‐WT but not in FGFR1‐15R and FGFR1‐25Ra expressing neurons. Furthermore, no activation of either PLC or STAT3 was observed in neurons overexpressing FGFR1‐WT or mutants after 30 min treatment with FGF‐2 (Fig. 4C).

### Trafficking and Subcellular Localization of FGFR1 Lysine Mutants

**Figure 5:**
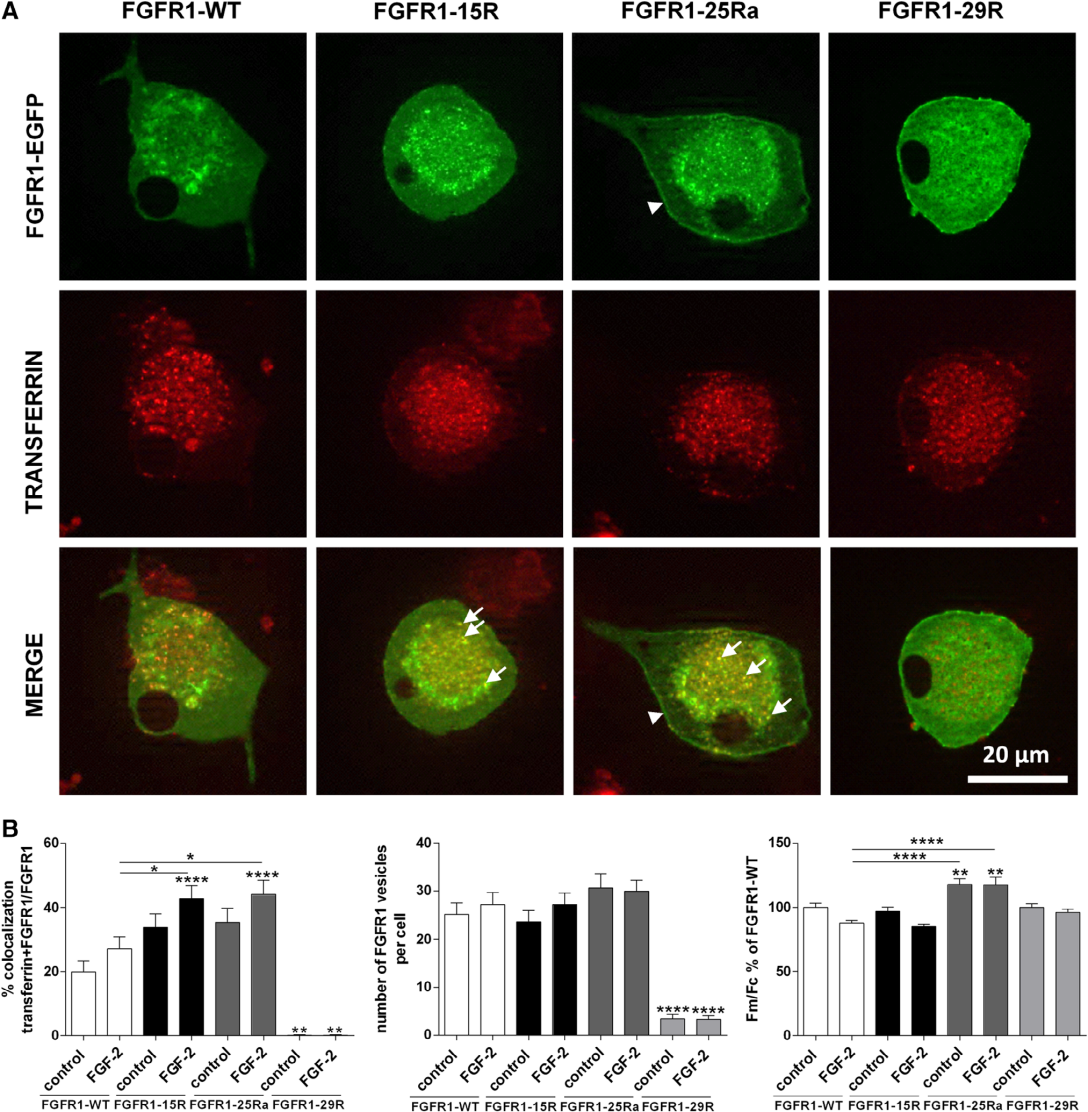
**(A)** Representative examples of colocalization of FGFR1‐WT‐EGFP, FGFR1‐15R‐EGFP, FGFR1‐25Ra‐EGFP, and FGFR1‐29R‐EGFP (green) with transferrin (red) after 30‐min treatment with FGF‐2 (100 ng/mL + 10 μg/mL heparan sulfate). Arrows indicate enhanced colocalization of FGFR1‐15R‐EGFP, and FGFR1‐25Ra‐EGFP with transferrin. Arrowheads label enhanced plasma membrane localization (Fm) of FGFR1‐25Ra‐EGFP. **(B)** Colocalization of FGFR1‐WT‐EGFP, FGFR1‐15R‐EGFP, FGFR1‐25Ra‐EGFP, and FGFR1‐29R‐EGFP with recycling endosomes visualized with transferrin conjugate in adult DRG neurons 30 min after treatment with FGF‐2 as revealed by ApoTome confocal‐like microscopy. Fluorescent intensity of the plasma membrane region (Fm) and average cytoplasmic fluorescence (Fc) were quantified and the Fm/Fc ratio was calculated for each cell after background correction. One‐way ANOVA with Tukey's posttest (mean ± SEM of four independent experiments with a total number of cells per group ≥70. *P < 0.1, **P < 0.01, and ****P < 0.0001 compared with FGFR1‐WT control). Abbreviation: DRG, dorsal root ganglia.

We then analyzed the recycling capabilities and cellular localization of EGFP‐fusion constructs expressing FGFR1‐WT, FGFR1‐15R, FGFR1‐25Ra, and FGFR1‐29R, which so far has only been investigated in non‐neuronal cells (Haugsten et al., 2008). In adult DRG neurons overexpressing FGFR1‐WT, about 20% of all FGFR1‐WT‐positive vesicles colocalized with transferrin (Fig. 5). This percentage increased for FGFR1‐15R and FGFR1‐25Ra 30 min after FGF‐2‐treatment as compared with FGFR1‐WT confirming previous data on enhanced recycling capabilities of FGFR1‐15R and FGFR1‐25Ra in tumor cells. The total number of FGFR1 positive vesicles was similar for FGFR1‐WT, FGFR1‐15R, and FGFR1‐25Ra indicating that the effects of FGFR1‐15R and FGFR1‐25Ra on elongative axon growth are not caused by different expression levels as compared with FGFR1‐WT (Fig. 5). The number of vesicles exhibiting FGFR1‐29R‐EGFP that is lysine‐deficient and not endocytosed is strongly reduced compared with FGFR1‐WT and mutants FGFR1‐15R and FGFR1‐25Ra. To test whether enhanced recycling of FGFR1‐15R and FGFR1‐25Ra leads to increased plasma membrane localization of these mutants, we investigated the plasma membrane fluorescence intensity (Fm) and compared it with the intensity of the cytoplasmic receptor fluorescence (Fc). Surface membrane localization of FGFR1‐25Ra was enhanced with or without FGF‐2, whereas surface membrane *versus* cytoplasma levels of FGFR1‐WT, FGFR1‐15R, and FGFR1‐29R were similar.

## DISCUSSION

Ligand‐induced activation of FGFR is followed by endocytosis and degradation in lysosomes, which leads to the termination of FGF signaling. Alternatively, receptors may be recycled from early endosomes back to the cell surface either directly or *via* the endocytic recycling compartment (Hoeller et al., 2005). RTKs remain active in the endosomal compartment and regulate signaling pathways differently as compared with their activation at the surface membrane (Sorkin and von Zastrow, 2002). FGFR1, ‐2, and ‐3 are mainly sorted to lysosomes for degradation, whereas FGFR4 is predominantly recycled back to the plasma membrane. FGFR1 reveals the fastest lysosomal degradation among all FGFRs, and the rate of recycling correlates well with the level of receptor ubiquitination that is much higher for FGFR1 than for FGFR4 (Haugsten et al., 2005).

The data presented in this work confirm our previous observations that mutating 25 lysine residues of FGFR1 results in enhanced recycling capabilities and promotion of elongative axon growth (Hausott et al., 2012). Interestingly, FGFR1‐15R with 14 remaining lysine residues was sufficient to strongly stimulate long‐distance axon regeneration by adult DRG neurons *in vitro* with or without FGF‐2. Although both mutants exhibited increased recycling capabilities, only FGFR1‐25Ra revealed enhanced surface membrane localization suggesting faster turnover of FGFR1‐WT and ‐15R as compared with FGFR1‐25Ra. However, pERK levels were lower than FGFR1‐WT at various time points after FGF‐2 treatment in cell bodies and axons indicating that mutant receptors do not act by overstimulating or prolonging ERK activation. Activation of AKT was not observed in neurons overexpressing mutant receptors suggesting that the most relevant signaling pathway for stimulating total axon outgrowth and, in particular, axon branching (PI3K/AKT) in adult peripheral neurons is not affected by the FGFR1‐15R or ‐25R mutants. Due to the limitations of quantitative immunofluorescence, however, the data have to be interpreted with caution. We are currently working on the establishment of signal reporter assays in living neurons to reveal more subtle differences in signaling at different subcellular locations (surface membrane, perinuclear recycling area, axons, growth cones, *etc*.). Unfortunately, protein blotting experiments were unsuccessful due to the low number of transfected neurons in our cultures.

Taken together, it appears likely that additional mechanisms—probably involving the C‐terminal tail of FGF receptors—are involved in the axon outgrowth response stimulated by forced expression of lysine‐deficient FGFR1 mutants such as enhanced interaction with cell adhesion molecules like NCAM that has been demonstrated to stimulate elongative axon outgrowth in an FGFR1‐dependent manner (Green et al., 1997; Saffell et al., 1997; Francavilla et al., 2007; Francavilla et al., 2009a; Chernyshova et al., 2011). This hypothesis is further supported by the present finding that the fusion of large proteins, such as EGFP, to the C‐terminus of the receptor abolishes the morphological effects on axon outgrowth.
